# Substrate specificity analysis of protein kinase complex Dbf2-Mob1 by peptide library and proteome array screening

**DOI:** 10.1186/1471-2091-6-22

**Published:** 2005-10-21

**Authors:** Angie S Mah, Andrew EH Elia, Geeta Devgan, Jason Ptacek, Mike Schutkowski, Michael Snyder, Michael B Yaffe, Raymond J Deshaies

**Affiliations:** 1 Department of Biology, California Institute of Technology, Pasadena, CA 91125, USA; 2 Howard Hughes Medical Institute, California Institute of Technology, Pasadena, CA 91125, USA; 3 Center for Cancer Research, Massachusetts Institute of Technology, Cambridge, MA 02139, USA; 4 Department of Biology, Massachusetts Institute of Technology, Cambridge, MA 02139, USA; 5 Division of Biological Engineering, Massachusetts Institute of Technology, Cambridge, MA 02139, USA; 6 Department of Molecular, Cellular, and Developmental Biology, Yale University, New Haven, CT 06520, USA; 7 Department of Molecular Biophysics & Biochemistry, Yale University, New Haven, CT 06520, USA; 8 JPT Peptide Technologies GmbH, Invalidenstrasse 130, 10115 Berlin, Germany, USA

## Abstract

**Background:**

The mitotic exit network (MEN) is a group of proteins that form a signaling cascade that is essential for cells to exit mitosis in *Saccharomyces cerevisiae*. The MEN has also been implicated in playing a role in cytokinesis. Two components of this signaling pathway are the protein kinase Dbf2 and its binding partner essential for its kinase activity, Mob1. The components of MEN that act upstream of Dbf2-Mob1 have been characterized, but physiological substrates for Dbf2-Mob1 have yet to be identified.

**Results:**

Using a combination of peptide library selection, phosphorylation of opitmal peptide variants, and screening of a phosphosite array, we found that Dbf2-Mob1 preferentially phosphorylated serine over threonine and required an arginine three residues upstream of the phosphorylated serine in its substrate. This requirement for arginine in peptide substrates could not be substituted with the similarly charged lysine. This specificity determined for peptide substrates was also evident in many of the proteins phosphorylated by Dbf2-Mob1 in a proteome chip analysis.

**Conclusion:**

We have determined by peptide library selection and phosphosite array screening that the protein kinase Dbf2-Mob1 preferentially phosphorylated substrates that contain an RXXS motif. A subsequent proteome microarray screen revealed proteins that can be phosphorylated by Dbf2-Mob1 in vitro. These proteins are enriched for RXXS motifs, and may include substrates that mediate the function of Dbf2-Mob1 in mitotic exit and cytokinesis. The relatively low degree of sequence restriction at the site of phosphorylation suggests that Dbf2 achieves specificity by docking its substrates at a site that is distinct from the phosphorylation site

## Background

In the budding yeast *Saccharomyces cerevisiae *the protein phosphatase Cdc14 must be activated to turn off mitotic Cdk activity for cells to exit mitosis. There are two groups of proteins that regulate Cdc14 activity, the Cdc14 early anaphase release (FEAR) network and the mitotic exit network (MEN) (reviewed in [1]).

There is one cyclin-dependent kinase (Cdk), Cdc28, that controls cell cycle progression in *S. cerevisiae *By associating with mitotic cyclins, Cdc28 promotes entry into mitosis. Cdc14 plays a critical role in inactivating mitotic Cdk activity, thereby promoting exit from mitosis. Cdc14 dephosphorylates Hct1/Cdh1 which can then target Clb2, the main mitosis-specific cyclin, for degradation [2-5]. Cdc14 also promotes the accumulation of Sic1, a Cdk inhibitor, by acting on Sic1 as well as transcriptional activator Swi5, which promotes transcription of *SIC1 *[3,6]. Together, the degradation of mitotic cyclins and the production of active Sic1 conspire to down-regulate Cdc28 activity and return the cell cycle to an interphase state.

Cdc14 is held in an inactive state in the nucleolus by its inhibitor, Net1 [7,8]. Cdc14 is tethered to Net1 throughout the cell cycle until the onset of anaphase, at which time it is released. The FEAR network and MEN both regulate Cdc14 release and therefore its activity (reviewed in [1,9]). The FEAR network initiates early anaphase release of Cdc14 from the nucleolus by promoting Net1 phosphorylation by mitotic Cdks, weakening the interaction between Cdc14 and Net1 [10]. The FEAR network consists of Esp1 (also known as separase), polo-like kinase Cdc5, the kinetochore protein Slk19, the nuclear protein Spo12, and its homologue Bns1. The action of these proteins is opposed by Pds1 (also known as securin) and the nucleolar replication fork block protein Fob1 [11-13]. However, it is still unclear how the factors that promote and restrain FEAR interact. Although the release of Cdc14 in early anaphase by the FEAR network is transient and insufficient for mitotic exit, exit is delayed when the FEAR network is compromised by mutation. One possible explanation is that the FEAR network weakens the Cdc14-Net1 interaction, enabling the MEN to more rapidly cause a sustained release of Cdc14. The FEAR network also has other mitotic functions that may play an important role in coordinating events during exit from mitosis (reviewed in [1,9]).

In contrast to the FEAR network, the MEN is essential for mitotic exit (reviewed in [1,9,14]). This pathway consists of the GTPase Tem1, the putative guanine-nucleotide exchange factor (GEF) Lte1, the two-component GTPase activating protein (GAP) Bub2-Bfa1, the protein kinases Cdc5, Cdc15, Dbf2, the Dbf2 binding protein Mob1, and the scaffolding protein, Nud1. Genetic and biochemical data have provided significant insight into how this signaling cascade is activated by the localization of its components. Bub2-Bfal binds and inhibits Tem1 at the spindle pole body (SPB) that enters the daughter cell during nuclear division. As the spindle elongates into the bud, Tem1 is presumably activated by Lte1, which is localized in the bud [15,16]. The activated GTP-bound form of Tem1 then somehow activates bound Cdc15, which then phosphorylates and activates the Dbf2-Mob1 kinase complex [17]. However, how activated Dbf2-Mob1 affects Cdc14 release from Net1 and mitotic exit is unknown.

In addition to their role in mitotic exit, Dbf2-Mob1 and the other MEN proteins function in cytokinesis. Dbf2 localizes to the SPB in anaphase as do Tem1, Cdc5, Cdc15, and Mob1 [15,16,18-21]. During late mitosis, Dbf2 and Mob1 migrate to the bud neck. Bud neck localization of Dbf2 and Mob1 are dependent on each other as well as the MEN proteins Cdc5, Cdc14, Cdc15, Nud1, and the septins Cdc12 and Cdc3 [19,22,23]. Several lines of evidence suggest that localization of MEN proteins to the bud neck is crucial for cytokinesis. Mutant *mob1*^*ts *^cells, as well as *tem1*Δ *net1-1 *and *cdc15*Δ *net1-1 *cells whose mitotic exit defects are bypassed by the *net1-1 *allele fail to undergo cytokinesis [7,23,24]. Interestingly, localization of Dbf2-Mob1 to the bud neck depends upon Cdc14 [23,25]. MEN-dependent release and activation of Cdc14 may help to ensure that mitotic exit occurs prior to cytokinesis.

The function of Dbf2-Mob1 in cytokinesis is unclear. Also unknown is how Dbf2-Mob1 ultimately leads to release of Cdc14 from the nucleolus during mitotic exit. To give us insight into these two key cell cycle processes, we sought to identify potential substrates and phosphorylation sites of Dbf2-Mob1. Here, we report the substrate specificity of Dbf2-Mob1 and a number of putative substrates that contain a Dbf2 phosphorylation motif and are phosphorylated by Dbf2-Mob1 *in vitro*.

## Results

### Determination of optimal peptide sequence motif phosphorylated by Dbf2-Mob1

To identify potential physiological substrates of Dbf2-Mob1, we first proceeded to determine an optimal substrate sequence by using an oriented degenerate peptide library technique [26]. Dbf2, a Ser/Thr kinase, was initially tested to determine whether there was a preference for phosphorylation on Ser or Thr residues. Degenerate peptide libraries containing either a fixed Ser residue, XXXXSXXXX, or a fixed Thr residue, XXXXTXXXX, were incubated with [γ-^32^P]ATP and Dbf2-Mob1 that was expressed in insect cells, purified, and activated by recombinant Cdc15. All amino acids except Cys, Ser, Thr, and Tyr are represented by X, where the last 3 residues were omitted to limit phosphorylation to the fixed Ser or Thr. The level of phosphorylation was determined by the amount of radioactive phosphate incorporated in the peptides. This analysis suggested that Dbf2-Mob1 had a preference for Ser phosphorylation over Thr (Table 1).

**Table 1:** Relative phosphate incorporation into peptide libraries by Dbf2-Mob1 kinase complex

**Peptide Library**	**Relative Phosphate Incorporation**
XXXXTXXXX	1
XXXXSXXXX	2
XXXXRXXSXXXX	16
XXXXSPXXXX	2

Recombinant baculovirus-derived ^FHH^Dbf2-^H6^Mob1^TM9 ^activated by baculovirus-derived Cdc15^His6 ^was used to screen different peptide libraries.

To determine the optimal peptide substrate for Dbf2-Mob1, we screened secondary libraries that contain a fixed residue in addition to the serine phosphorylation site. The rationale for this is articulated in Songyang et. al. [27]. We chose to examine a library with an R fixed at -3 (X4RX2SX4), because Dbf2 resides on the 'AGC' branch of the protein kinase family tree [28]. Other kinases on this branch, including AKT [26] and PKA [27] preferentially phosphorylate substrates with an R at -3. As a control, we also examined a library with the sequence X4SPX4. The X4RX2SX4 library was found to incorporate 8 times more phosphate than the X4SPX4 library (Table 1). As a result, the X4RX2SX4 library was used to determine an optimal Dbf2-Mob1 substrate motif. Sequencing a pool of Dbf2-phosphorylated peptides enriched from the RS library revealed a strong preference for Ile and Phe at the -2 and -1 positions, respectively (Table 2). There was a moderate selection for Met at both the -4 and +1 positions (Table 2). The predicted optimal consensus motif for Dbf2-Mob1 substrates was determined to be MRIFSM.

**Table 2:** Amino acids selected in a peptide library screen for Dbf2-Mob1 substrates

**-7**	**-6**	**-5**	**-4**	**-3**	**-2**	**-1**	**0**	**+1**	**+2**	**+3**	**+4**
X	X	X	M(1.4)	**R**	**I(2.8)**	**F(2.8)**	**S**	**M(1.4)**	X	X	X
			F(1.1)		**V(1.7)**	**V(1.6)**		I(1.1)			
					**H(1.7)**	I(1.3)		L(1.1)			
					M(1.2)	M(1.1)		V(1.1)			

Activated ^FHH^Dbf2-^H6^Mob1^TM9 ^was used to screen the peptide library with the sequence X-X-X-X-R-X-X-S-X-X-X-X. Relative selectivities for amino acids are indicated in parentheses. Bold letters indicate amino acids that are strongly selected; X indicates no selectivity. The one-letter amino acid code is used.

### Optimal sequence phosphorylation efficiency

**Figure 1:**
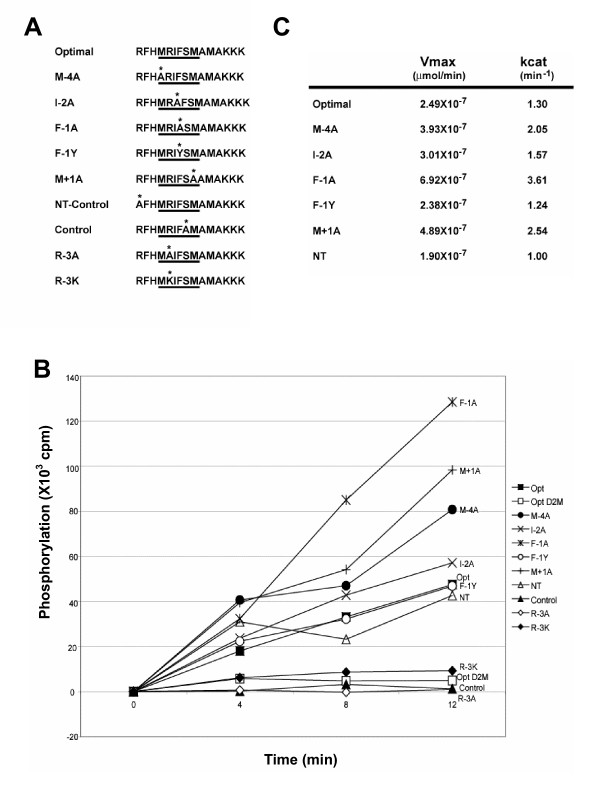
**Dbf2-Mob1 peptide substrate requires arginine at position -3**. **(A) **Synthetic peptides based on the predicted optimal substrate of Dbf2-Mob1. The underlined residues represent the predicted preferred amino acids for Dbf2-Mob1 substrate specificity; the asterik denotes the single amino acid substitution. **(B) **The various peptides denoted in (A) at a concentration of 250 μM were treated with ~13 ng of ^FHH^Dbf2 bound to ^H6^Mob1^TM9^. Aliquots of the kinase reaction were quenched at the indicated timepoints to determine the amount of phosphorylation by liquid scintillation. The Optimal peptide was also treated with the kinase-inactive ^FHH^Dbf2(N305A)-^H6^Mob1^TM9 ^complex, as denoted by D2M. Similar results were obtained in 4 independent experiments. **(C) **Using the conditions in (B), K_m _and V_max _was determined for each peptide with the exception of R-3K, R-3A, negative control, and the Optimal peptide treated with kinase-inactive ^FHH^Dbf2(N305A)-^H6^Mob1^TM9^, due to low phosphorylation.

To evaluate the contribution of each residue in the predicted optimal consensus motif, we synthesized a peptide based on the consensus motif, as well as a set of variants in which each position was substituted by an alanine residue (Figure 1A). Peptides with Tyr at -1 (F-1Y) and Lys at -3 (R-3K) were also synthesized to determine whether the selection of Phe at position -1 could be replaced by another bulky residue like Tyr, or if the Arg at position -3 could be substituted with the similarly charged Lys. The NT-Control peptide contains a substitution in the Arg residue that lies outside of the predicted consensus motif to determine whether there is a selection at that position. Finally, we generated a negative control peptide that contains the consensus except that the Ser phosphorylation site was replaced with Ala.

The synthetic peptides were treated with Dbf2-Mob1 *in vitro *for 0, 4, 8 or 12 minutes and the amount of phosphorylation was determined (Figure 1B). As a negative control, the optimal consensus peptide was treated in parallel with the kinase-inactive Dbf2(N305A)-Mob1 that had undergone the same treatment with Cdc15 as the wild type Dbf2-Mob1 complex (Opt D2M). As expected, the negative control peptide, as well as the R-3A peptide both had negligible levels of phosphorylation (Figure 1B; Inh, R-3A). The optimal peptide treated with kinase inactive Dbf2(N305A)-Mob1 was also negligibly phosphorylated (Figure 1B; Inh). For the remaining peptides in which measurable levels of phosphorylation were detected, the V_max _and k_cat _for Dbf2-Mob1 were calculated (Figure 1C).

There did not appear to be selection for Ile at the -2 position nor selection for the Arg in the -7 position outside of the consensus motif, as both mutant peptides were phosphorylated to a similar degree as the optimal peptide (Figure 1B; I-2A, NT, Opt), with similar V_max_and k_cat _values for Dbf2-Mob1 (Figure 1C). Mutations of the bulkier groups at positions -1, -4, and +1 to Ala actually increased the amount of phosphorylation of the peptides (Figure 1B; F-1A, M-4A, M+1A), increasing the V_max _and k_cat _values for Dbf2-Mob1 (Figure 1C). This was surprising because the peptide library screen had indicated a strong selection for Phe at position -1. However, the FLAG peptide used to elute Dbf2-Mob1 may have influenced the selection of phosphopeptides during the screening procedure. Interestingly, the substitution of Arg by Lys in position -3 decreased the amount of peptide phosphorylation to a level comparable to the negative controls (Figure 1B; R-3K). Taken together, these results revealed a preference for non-bulky residues proximal to the Ser phosphorylation site and that the critical Arg required for substrate phosphorylation cannot be substituted with Lys – at least in the context of a peptide substrate.

To confirm the results of the peptide library screen by an independent method, we screened a phosphosite array with active and kinase-dead Dbf2-Mob1 in the presence of [^32^P]-ATP. The phosphosite array contains 2296 peptides that correspond to annotated sites of phosphorylation in the human proteome [29]. Replicate experiments were performed, and the 'hits' that were obtained with kinase-dead Dbf2-Mob1 were subtracted. Analysis of the top 20 peptide substrates for active Dbf2 (based on intensity of incorporated label) from the first experiment revealed that 18/20 contained a serine phosphorylation site (the remaining two contained threonine), whereas all twenty possessed an R at -3. In the replicate experiment, all eight candidates had an RXXS motif. The only other bias revealed in this analysis – albeit a modest one – was a general preference for R in positions N-terminal to the phosphorylation site. Thus, two different methods – a library-based selection for phosphopeptides and a screen of a phosphopeptide array – suggest that Dbf2 phosphorylates serines spaced three amino acid downstream of an arginine.

### Proteome array screen identifies *in vitro *Dbf2-Mob1 substrates

The relatively low sequence complexity of the Dbf2 phosphorylation motif diminished the power of using genome-wide bioinformatics screens to identify putative substrates. Accordingly, we carried out a proteome array screen to identify putative yeast substrates. Proteome chips spotted with ~4,400 different glutathione-S-transferase (GST) fusion proteins purified from yeast were probed with activated Dbf2-Mob1, kinase-inactive Dbf2(N305A)-Mob1, and buffer alone. After taking into account the proteins phosphorylated in the negative controls, 67 proteins were determined to be putative Dbf2-Mob1 substrates (Table 3).

**Table 3:** Putative Dbf2-Mob1 substrates from proteome chip screen

**ORF Name**	**Common Name**	**Slide Signal (after normalization)**
YJR060W	CBF1	118.4224
YAL051W	OAF1	81.8589
YBR138C	HDR1	8.9063
YOL012C	HTZ1	4.0738
YMR165C	SMP2	3.2182
YPL150W		3.1545
YDR226W	ADK1	3.1016
YMR229C	RRP5	2.6954
YBR118W	TEF2	2.619
YJL108C	PRM10	2.5802
YPR091C		2.5784
YKL168C	KKQ8	2.5013
YNL101W	AVT4	2.1486
YBR285W		1.9355
YMR239C	RNT1	1.593
YNR047W		1.4015
YJL076W	NET1	1.3521
YNL155W		1.2912
YNR006W	VPS27	1.1429
YIL135C	VHS2	1.116
YMR184W		1.1144
YDL220C	CDC13	1.0274
YBR108W		1.0216
YDL019C	OSH2	0.9962
YOR362C	PRE10	0.9895
YNL284CA	MRPL10	0.9442
YKL077W		0.8905
YDR134C		0.8135
YDL002C	NHP10	0.8102
YMR072W	ABF2	0.7585
YGR038CA		0.7292
YOR228C		0.6681
YKL140W	TGL1	0.6321
YDL070W	BDF2	0.6162
YCR105W	ADH7	0.6146
YBL106C	SRO77	0.5988
YNL125C	ESBP6	0.5694
YHR182W		0.4773
YJL213W		0.4755
YDR299W	BFR2	0.47
YPL211W	NIP7	0.4521
YML037C		0.4503
YDR171W	HSP42	0.442
YOL104C	NDJ1	0.3611
YKL146W	AVT3	0.3592
YGL245W		0.2863
YIL010W	DOT5	0.2606
YNL007C	SIS1	0.2239
YHL021C		0.2094
YMR196W		0.2089
YJR142W		0.2005
YGR220C	MRPL9	0.1976
YLR177W		0.1626
YJL211C		0.1594
YML035C	AMD1	0.1384
YGL105W	ARC1	0.1332
YGR264C	MES1	0.1328
YPL257WA		0.1302
YBL024W	NCL1	0.1294
YJR094WA		0.1141
YLR007W	NSE1	0.0988
YLR303W	MET17	0.0985
YGR223C		0.0837
YKR022C		0.0806
YLL008W	DRS1	0.0702
YFR033C	QCR6	0.042
YLR004C		0.0228

Activated ^FHH^Dbf2-^H6^Mob1^TM9 ^was used to screen the yeast proteome chip. Of 86 proteins phosphorylated by ^FHH^Dbf2-^H6^Mob1^TM9^, 67 were determined to be putative substrates after taking into account proteins that were phosphorylated in the control slides treated with the kinase-inactive ^FHH^Dbf2(N305A)-^H6^Mob1^TM9^.

To confirm that the proteins identified in the proteome array screen could be phosphorylated by Dbf2-Mob1 as opposed to being bound to Dbf2 substrates, we performed further analyses on the 25 proteins with the highest incorporation signal relative to protein amount (relative protein amounts were determined by anti-GST immunoblot of the proteome chip). Interestingly, three or more copies of this motif were found in 16, or 64%, of the proteins in the list (Table 4), compared to only 29% of the proteins encoded in the yeast genome. This enrichment for proteins with 3 or more copies of the RXXS motif is highly significant (p = 1.2 × 10^-4^; G. Kleiger, unpublished data).

**Table 4:** Proteins with highest phosphorylation signal from proteome chip screen for Dbf2-Mob1 substrates

**ORF Name**	**Common Name**	**Slide Signal (after normalization)**	**MW (kDa)**	**# of R-3 Sites**
YJR060W	CBF1	118.4224	39	3
YAL051W	OAF1	81.8589	121	3
YBR138C	HDR1	8.9063	61	2
YOL012C	HTZ1	4.0738	14	1
YMR165C	SMP2	3.2182	95	7
YPL150W		3.1545	100	16
YDR226W	ADK1	3.1016	24	0
YMR229C	RRP5	2.6954	193	5
YBR118W	TEF2	2.619	50	0
YJL108C	PRM10	2.5802	41	0
YPR091C		2.5784	87	3
YKL168C	KKQ8	2.5013	84	14
YNL101W	AVT4	2.1486	80	7
YBR285W		1.9355	17	0
YMR239C	RNT1	1.593	54	2
YNR047W		1.4015	100	21
YJL076W	NET1	1.3521	128	11
YNL155W		1.2912	31	1
YNR006W	VPS27	1.1429	71	4
YIL135C	VHS2	1.116	48	11
YMR184W		1.1144	22	3
YDL220C	CDC13	1.0274	105	3
YBR108W		1.0216	93	7
YDL019C	OSH2	0.9962	146	6
YOR362C	PRE10	0.9895	32	0

Of the 67 putative substrates, the 25 putative substrates with the highest relative amount of phosphorylation signal (amount of signal relative to protein expression) as listed were chosen for further study. MW: predicted molecular weight, # or R-3 Sites: number of RXXS motifs

**Figure 2:**
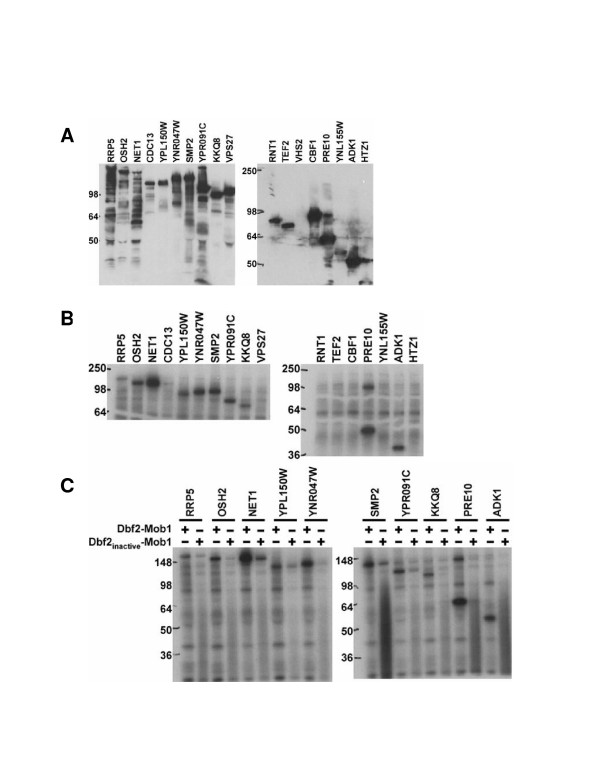
**Yeast proteins phosphorylated by Dbf2-Mob1. (A) **Of the 25 proteins with the highest phosphorylation signal as shown in Table 4, 22 of these genes were TAP-tagged in the Open Biosystems TAP-tagged yeast library. The TAP-tagged proteins were immunoprecipitated with IgG sepharose beads from asynchronous cultures, fractionated on SDS-PAGE and immunoblotted with anti-TAP. Of the 22 strains, 5 did not have detectable protein expression, such as VHS2 as shown. **(B) **The TAP-tagged proteins expressed in (A) were treated with ^FHH^Dbf2-^H6^Mob1^TM9 ^in the presence of [γ-^32^P]ATP, fractionated on SDS-PAGE and detected by autoradiography. **(C) **The TAP-tagged proteins phosphorylated by ^FHH^Dbf2-^H6^Mob1^TM9 ^in (B) were treated with either ^FHH^Dbf2-^H6^Mob1^TM9 ^or the kinase inactive ^FHH^Dbf2(N305A)-^H6^Mob1^TM9 ^in the presence of [γ-^32^P]ATP, fractionated on SDS-PAGE and detected by autoradiography.

TAP-tagged strains were obtained for 22 of the 25 top candidates. TAP-tagged alleles for three of the genes (YJL108C, YBR285W, and YBR108W) were not available, and therefore these candidates were not subjected to further analysis. Asynchronous cultures of the 22 TAP-tagged strains were grown and the TAP-tagged proteins immunoprecipitated with IgG sepharose and analyzed by Western blotting (Figure 2A). Candidates were determined to be phosphorylated if a radioactive signal was detected at the molecular weight predicted for the tagged protein. Of the 22 strains in which immunoprecipitations were performed, YBR138C (HDR1), YAL051W (OAF1), YNL101W (AVT4), YIL135C (VHS2), and YMR184W did not have detectable protein expression. The 17 TAP-tagged proteins that were expressed and purified were used in Dbf2-Mob1 kinase assays (Figure 2B). Of the 17, 10 were determined to be phosphorylated. To determine whether the phosphorylation of these proteins was specific to Dbf2-Mob1, rather than due to a co-precipitating protein kinase or residual Cdc15 used to activate Dbf2-Mob1, kinase assays were performed using kinase-inactive Dbf2(N305A-Mob1) as a negative control (Figure 2C). In all cases, there was a strong decrease in incorporation when kinase-inactive Dbf2-Mob1 was used. These results suggest that the proteins identified by the proteome chip screen were indeed in vitro substrates of Dbf2-Mob1.

In attempts to confirm whether any of the Dbf2-Mob1 substrates identified by our analyses are true physiological substrates of this complex, we took four approaches. First, we examined whether any of the substrates undergo a molecular weight shift upon their phosphorylation by Dbf2-Mob1, which might serve as a simple diagnostic to evaluate phosphorylation *in vivo*. Next, we immunoprecipitated each protein from yeast cells via the TAP tag, and immunoblotted for Mob1 to determine if the putative substrates were associated with the Dbf2 complex. Third, we queried the yeast GFP localization database, to see if any of the candidates display SPB or bud neck localization characteristic of Dbf2-Mob1. Finally, we searched a database of 700 mapped yeast phosphorylation sites to see if any of them reside in our candidate substrates. Unfortunately, none of these efforts yielded a positive result (A. M., unpublished data). This experience highlights that validation of putative protein kinase substrates identified by proteome chip analysis may require considerable investment in the mapping of *in vivo *phosphorylation sites.

## Discussion

The substrate used to test Dbf2-Mob1 kinase activity, histone H1, is a commonly used artificial substrate for many protein kinases. We wanted to find physiological protein substrates of Dbf2-Mob1. To do so, we first sought to define the optimal phosphorylation site motif for Dbf2-Mob1 substrates. Peptide library screening revealed the putative optimal substrate motif to be MRIFSM (Figure 1A). However, when we tested each residue in *in vitro *Dbf2-Mob1 peptide kinase assays, the only substitutions that diminished incorporation were swapping the Arg in position -3 for Ala or Lys (Figure 2B). The latter result was unexpected, because Ndr1, a human homologue of Dbf2, was proposed to phosphorylate sequences with either Lys or Arg in the -3 position [30]. Although Dbf2 exhibits a strong requirement for an R at -3, it displays remarkably little bias for any other position adjacent to the phosphorylation site. A similar result was obtained by performing a screen of a phosphosite array composed of peptides that correspond to annotated sites of phosphorylation in the human proteome.

The proteins identified by proteome chip screening gave further evidence that the RXXS motif serves as a substrate for Dbf2-Mob1 phosphorylation, as 80% of the top 25 proteins that were identified in the proteome chip analysis contained this motif. Of these proteins, 17 were tested further and 10 of these were identified as *in vitro *substrates, 8 of which have the RXXS motif. Pre10 and Adk1 were phosphorylated by Dbf2-Mob1 but not its kinase inactive form despite not having the RXXS motif (Figure 2C; Table 4). One reason may be that both proteins are immunoprecipitated at high levels and therefore may serve as non-specific substrates of Dbf2-Mob1.

Recently, the results of a systematic proteome array analysis of yeast protein kinases have been posted online (J. Ptacek *et al., *submitted). There were a few minor discrepancies between the results posted online for Dbf2-Mob1 and those of Table 3, with the exception of Cbf1, Oaf1, and Htz1. These 3 proteins were within the top 4 proteins that gave the highest signal in our original data set (Table 3) yet were not in the data set posted online. The discrepancies were based on the methodology for identifying positive signals. In our original data set, the results were obtained by computer analysis. Further visual analysis to confirm positives was performed for the data available online. Upon visual confirmation, Cbf1, Oaf1, and Htz1 were determined to be incorrectly identified by computer analysis (J. Ptacek, personal communication). Our *in vitro *analysis confirmed that two of these proteins (Oaf1 was not tested because a TAP-tagged strain was not available) were indeed negatives as neither Cbf1 nor Htz1 were found to be phosphorylated by Dbf2-Mob1 (Figure 2B).

## Conclusion

We have determined that protein kinase Dbf2-Mob1 has a preference for phosphorylating peptides and proteins that bear one or more RXXS motifs. Although there is strong selection for the R at -3, there is remarkably little selection at any other position, suggesting that Dbf2 achieves specificity by docking its substrates at a site that is distinct from the phosphorylation site.

## Methods

### Purification and activation of Dbf2-Mob1 kinase complex

^FlagHis6HA3^Dbf2 (^FHH^Dbf2) was co-immunoprecipitated with ^His6^Mob1^TEVmyc9 ^(^H6^Mob1^TM9^) from Hi5 insect cells as previously described [17]. To activate ^FHH^Dbf2-^H6^Mob1^TM9^, the protein complex bound to anti-FLAG M2 beads (Sigma) was incubated with baculovirus-expressed Cdc15^His6 ^in the presence of kinase buffer containing 50 mM HEPES (pH 7.5), 5 mM MgCl_2_, 2.5 mM MnCl_2_, 5 mM β-glycerophosphate, 1 mM DTT, and 1 mM ATP for 30 min at room temperature. The beads were washed three times with buffer containing 50 mM Tris (pH 7.6), 150 mM NaCl, 0.2% Triton X-100 to remove ATP and Cdc15^His6^. ^FHH^Dbf2-^H6^Mob1^TM9 ^was then eluted from the beads with 1 μg/ml FLAG peptide (Sigma) in Dbf2 kinase buffer (DKB) containing 50 mM Tris (pH 7.4), 60 mM potassium acetate, 10 mM MgCl_2_, 1 mM DTT, and 10 μM ATP for four hours at 4°C. The same procedure was used to produce ^FHH^Dbf2(N305A)-^H6^Mob1^TM9^, the kinase inactive point mutant of Dbf2. The eluted active or inactive ^FHH^Dbf2-^H6^Mob1^TM9 ^was used for subsequent assays.

### Peptide library screening

Baculovirus-derived ^FHH^Dbf2-^H6^Mob1^TM9 ^was used for peptide library screening. Peptide library screening and data analysis were performed as previously described [26,31,32]. Briefly, the X4RX2SX4 peptide library was used for screening. This library consists of peptides with the general sequence MAXXXXRXXSXXXXAKKK, where X represents all amino acids except Cys, Ser, Thr, and Tyr. The total library is predicted to contain 1.1 × 10^12 ^distinct sequences. Peptides (~1 mg) were incubated with ^FHH^Dbf2-^H6^Mob1^TM9^, 100 μM unlabeled ATP, and tracer amounts of [γ-^32^P]ATP at 30°C for 2 h until ~0.5–1% of peptides were phosphorylated. ATP was removed by DEAE-dextran column and a ferric-iminodiacetic acid (IDA) column was then used to separate the phosphopeptides from non-phosphorylated peptides. The phosphopeptides were then sequenced in batch by automated Edman degradation. Data analysis was as described [32]. Selectivity values refer to the relative preference for an amino acid at a given degenerate position, based on the amount of each amino acid recovered at that position compared to the amount in the starting library [31].

### Peptide kinase assays

Synthetic peptides (Abgent) were used as substrates for ^FHH^Dbf2-^H6^Mob1^TM9 ^kinase assays. Reactions containing 250 μM of peptide substrate and ~13 ng of ^FHH^Dbf2 bound to ^H6^Mob1^TM9 ^were incubated in the presence of 30 μl of DKB and 2 μCi [γ-^32^P]ATP at room temperature. Reaction aliquots were terminated at indicated timepoints by addition of 10 μl of stop solution (8 N HCl, 1 mM ATP). Phosphate incorporation was determined by spotting reactions on P81-phosphocellulose paper (Whatman), washing with 0.5% phosphoric acid, air-drying the filters, and then quantifying the bound radioactivity by scintillation counting. For each individual peptide, values were normalized to time zero.

### Phosphosite array screening

Baculovirus-derived ^FHH^Dbf2-^H6^Mob1^TM9 ^was used for phosphosite array screening. Peptide microarrays displaying phosphosite derived peptides and data analysis were performed as previously described [29,33,34]. Briefly, 2296 peptides derived from human phosphosites (annotated phosphosite in the middle position of 13mer peptide) were printed in triplicates onto aldehyde-modified glass slides [29]. ^FHH^Dbf2 bound to ^H6^Mob1^TM9 ^was incubated in the presence of 300 μl of DKB and 20 μCi [γ-^32^P]ATP at room temperature for 4 hours. Slide was washed 5 times with 25 mL 0.1 M phosphoric acid for 3 minutes followed by washings with 25 mL deionised water. Finally, microarrays were washed with 25 mL methanol and dried at room temperature. Detection of incorporated radioactivity was performed by exposition of the microarrays for 8 hours to a BAS-MS imaging plate (Fuji Photo Film Co., Ltd., Japan) followed by readout with a FLA-3000 Phosphor Imager (Fuji, Japan). Data evaluation was carried out using ArrayPro software (Media Cybernetics, Silver Spring, MD, USA). Similar experiments with the kinase dead mutant were performed. Signals with high intensity in the active kinase experiment but low intensity in the kinase dead control were considered as peptide substrates specific for the Dbf2-Mob1 complex.

### Proteome chip assays

Yeast proteome microarrays were prepared as previously described [35]. Overexpressed GST-tagged yeast proteins were purified from ~4,400 yeast strains and spotted on slides. To determine the optimal amount of kinase to use for probing proteome chips, we performed trial assays as described (J. Ptacek *et al., *submitted). Multiple dilutions of ^FHH^Dbf2-^H6^Mob1^TM9 ^and kinase inactive ^FHH^Dbf2(N305A)-^H6^Mob1^TM9 ^(~20 ng/μl of Dbf2) in DKB buffer containing 2 μl [γ-^33^P]ATP were used on trial proteome chips before using on the full proteome array. The full proteome array was probed with 4 μl ^FHH^Dbf2-^H6^Mob1^TM9 ^in 200 μl of DKB supplemented with [γ-^33^P]ATP. As a control, ^FHH^Dbf2(N305A)-^H6^Mob1^TM9 ^was used to probe the proteome array. To normalize the background signal, 2 μl of the kinase-inactive complex in 200 μl DKB supplemented with [γ-^33^P]ATP was used. To control for autophosphorylated proteins, the proteome array was probed with 200 μl of DKB supplemented with [γ-^33^P]ATP. Proteome chips were assayed in duplicate in each case. Data analysis was performed as described (J. Ptacek *et al*., submitted). Briefly, signals were analyzed by a computer algorithm designed to normalize background and identify signals as positive if 3 of 4 spots (each protein is spotted twice on each slide and each kinase or control was tested on 2 slides) were 2 standard deviations above background and the fourth spot was 1.5 standard deviations above background.

### Immunoprecipitations and kinase assays of TAP-tagged proteins

Yeast strains containing TAP-tagged genes (Open Biosystems) were grown to OD_600 _~2.0 in 25 mL of YPD. Cells were harvested by centrifugation and washed in buffer containing 150 mM NaCl and 50 mM Tris (pH 7.4). Cells were then resuspended in 600 μl lysis buffer containing 150 mM NaCl, 50 mM Tris (pH 7.4), 2 mM EDTA (pH 8.0), 1% Triton-X 100, 10% glycerol, 2 mM DTT, 5 μg/ml aprotinin, 5 μg/ml pepstatin, 5 μg/ml chymostatin, 5 μg/ml leupeptin, 0.5 mM AEBSF, 1 mM PMSF, 10 mM NaF, 60 mM β-glycerophosphate, 10 mM sodium pyrophosphate, 2 mM sodium vanadate). An equal volume of glass beads was added. The cells were then lysed by 4 cycles of vortexing (ThermoSavant FastPrep) at 4°C for 45 s at setting 5.5 alternating with cycles of icing samples for 1 min. Lysates were clarified by centrifugation then added to 60 μl IgG sepharose beads (Amersham) for 1 h at 4°C on a rotator. Beads were then washed 3 times with lysis buffer, twice with buffer containing 150 mM NaCl, 50 mM Tris (pH 7.4), 2 mM EDTA (pH 8.0), 1% Triton-X 100, 10% glycerol, and 2 mM DTT, and a final wash with buffer containing 150 mM NaCl and 50 mM Tris (pH 7.4). Immunoprecipitated TAP-tagged proteins were analyzed by SDS-PAGE and detected by Western blotting using the primary antibody anti-TAP (Open Biosystems) followed by goat anti-rabbit horseradish peroxidase (HRP)-conjugate (Bio-Rad), and ECL. For kinase assays, TAP-tagged proteins bound to 20 μl beads were washed with DKB then incubated with ^FHH^Dbf2-^H6^Mob1^TM9 ^or ^FHH^Dbf2(N305A)-^H6^Mob1^TM9 ^(~13 ng of Dbf2) with 2 μCi [γ-^32^P]ATP for 30 min at room temperature. Kinase reactions were stopped by addition of 2X SDS-PAGE sample buffer, fractionated on SDS-PAGE and detected by autoradiography.

## List of abbreviations

MEN, mitotic exit network; FEAR, Cdc14 early anaphase release; GEF, guanine-nucleotide exchange factor; GAP, GTPase activating protein; SPB, spindle pole body; GST, gluathione-S-transferase; IDA, iminodiacetic acid; FHH, FlagHis6HA3; H6, His6; TM9, TEV-myc9; DKB, Dbf2 kinase buffer

## Authors' contributions

ASM performed the peptide kinase assays and analysis, the immunoprecipitations and kinase assays of the TAP-tagged strains, preparation of recombinant Dbf2-Mob1 complexes used throughout the work, and preparation of the manuscript. AEHE carried out the peptide library screening and analysis. GD and JP carried out the proteome chip studies. MS performed the phosphosite array screening. MS, MBY, and RJD contributed to the experimental design, analysis, and interpretation.
